# Complex Population Response of Dorsal Putamen Neurons Predicts the Ability to Learn

**DOI:** 10.1371/journal.pone.0080683

**Published:** 2013-11-14

**Authors:** Steeve Laquitaine, Camille Piron, David Abellanas, Yonatan Loewenstein, Thomas Boraud

**Affiliations:** 1 UMR 5293, University of Bordeaux, Bordeaux, France; 2 UMR 5293, CNRS, Bordeaux, France; 3 Department of Neurobiology, Edmond and Lily Safra Center for Brain Sciences Interdisciplinary Center for Neural Computation and Center for the Study of Rationality, Hebrew University of Jerusalem, Jerusalem, Israel; 4 CHU Bordeaux, Bordeaux, France; Inserm, France

## Abstract

Day-to-day variability in performance is a common experience. We investigated its neural correlate by studying learning behavior of monkeys in a two-alternative forced choice task, the two-armed bandit task. We found substantial session-to-session variability in the monkeys’ learning behavior. Recording the activity of single dorsal putamen neurons we uncovered a dual function of this structure. It has been previously shown that a population of neurons in the DLP exhibits firing activity sensitive to the reward value of chosen actions. Here, we identify putative medium spiny neurons in the dorsal putamen that are cue-selective and whose activity builds up with learning. Remarkably we show that session-to-session changes in the *size* of this population and in the intensity with which this population encodes cue-selectivity is correlated with session-to-session changes in the *ability* to learn the task. Moreover, at the population level, dorsal putamen activity in the very *beginning* of the session is correlated with the performance at the *end* of the session, thus predicting whether the monkey will have a "good" or "bad" learning day. These results provide important insights on the neural basis of inter-temporal performance variability.

## Introduction

According to the widely-held “hot hand” belief, athletes have periods in which their performance is significantly better than could be expected on the basis of their past record and periods in which their performance is significantly worse (persistence in failure). Similarly, it is widely believed that our every-day performance fluctuates between days, a phenomenon often referred to as “good days” and “bad days” in popular terminology. Whether or not “good days” and “bad days” exist in professional sports is a hotly debated subject [1-3]. It is also well known that abnormal fluctuations in performance in cognitive tasks is an early symptom of cognitive impairment in neurodegenerative disorders [4,5] and is also linked to psychiatric conditions such as impulsivity and pathological gambling [6,7]. In this study we used a two alternative forced choice task, the two armed-bandit task [8-12], to characterize fluctuations in two monkeys’ ability to learn the mapping between cues and probabilistic reward outcomes. To identify neural correlates of these fluctuations, we recorded from multiple neurons the sensorimotor and motor planning areas of the striatum (in dorsal putamen) of monkeys while they performed the task. The posterior dorsal putamen (dorsolateral striatum in rodents) is essential for efficiently solving repetitive goal-directed tasks [13]. In particular, it has been shown to be critical to the formation of habits after extensive exposures to cues in rodents [15,16], non-human primates [14] and humans [17]. Model-free reinforcement learning, the dominant computational theory of instrumental learning, asserts that habits are controlled by rigid cached mappings between cues and responses that are shaped by the history of rewards (action values) associated with each response [18]. This theory is supported by a bulk of studies that have identified neural correlates of action values in various regions of the striatum during learning [19-21]. However it is not clear if putamen contributes to habits via action values only. Here we examine the spiking activity of neurons classified as striatal projection neurons and show that learning is associated with changes in the response pattern of this population of neurons: (1) inline with previous studies, we identify a subpopulation of striatal projection neurons that directly encodes preference for cues ; (2) the size of this subpopulation and the preference encoding magnitude are correlated with the ability of the monkey to learn the task ; 3) remarkably, putamen’s population activity in the first three trials of the block predicts monkeys’ ability to learn the task. 

## Materials and Methods

### Animal and surgery

Data were obtained from two female macaque monkeys (Macaca mulata) weighing 2.5 and 4 kg. All experiments were performed during daylight hours. Although food was available *ad libitum*, the monkeys were kept under water restriction to increase their motivation during the learning task and recording sessions. A veterinarian skilled in the healthcare and maintenance of non-human primates supervised all aspects of animal care. Surgical and experimental procedures were performed in accordance with the Council Directive of 24 November 1986 (86/609/EEC) of the European Community and the National Institute of Health *Guide for the Care and Use of Laboratory Animals*. 

### Behavioral Task

**Figure 1:**
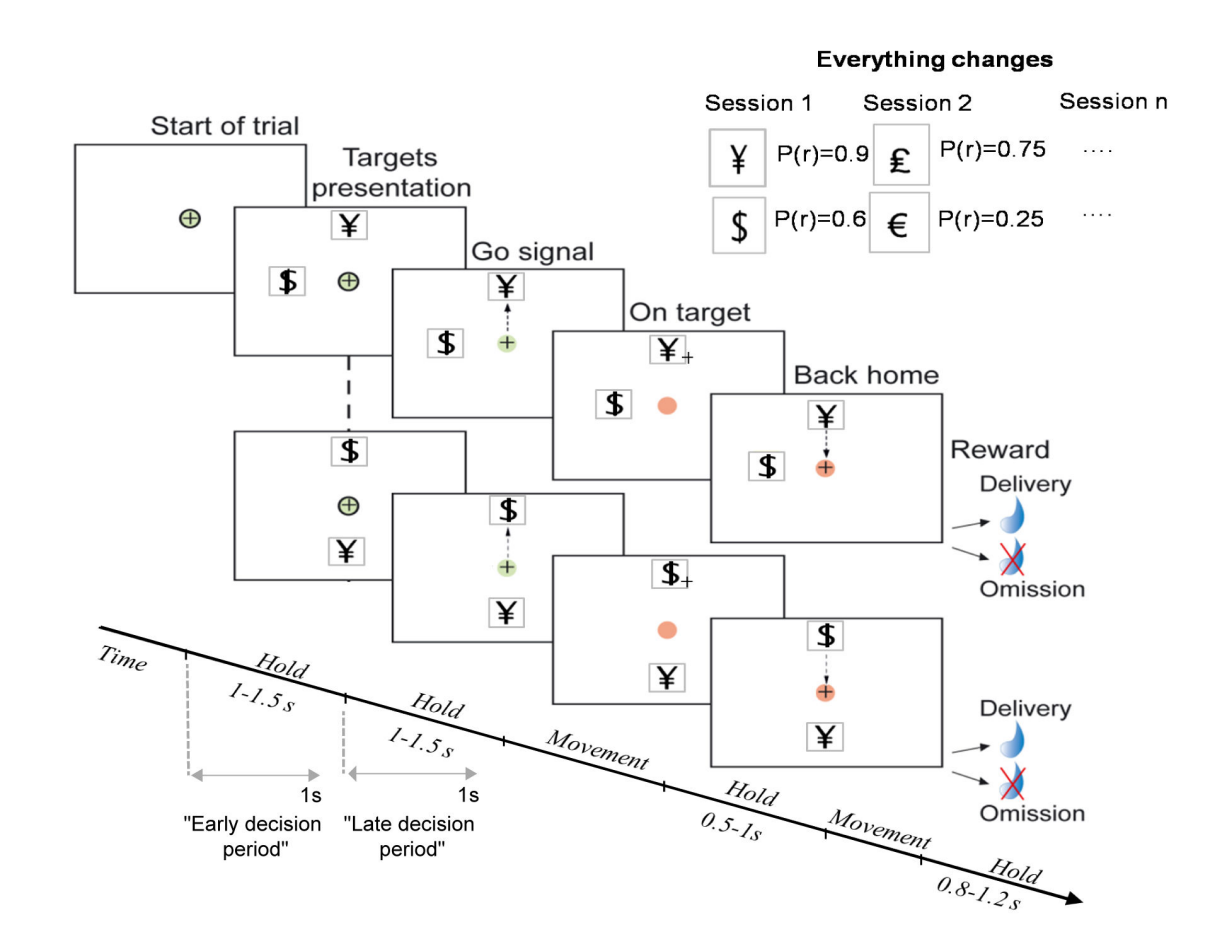
The task. In each trial, two cues were displayed simultaneously in two out of four randomly chosen possible positions on the screen. The monkey signaled its choice by moving the cursor to one of the cues and was rewarded by 300 μl of fruit juice with a predefined fixed probability that depended on the choice. We used 3 different pairs of reward probabilities for the two cues: (0.9 vs. 0.6), (0.75 vs. 0.25) and (0.67 vs. 0.33). Top right shows example combinations of displayed cues during different sessions.

The task was monitored using Labview (National Instruments, Austin, TX). Monkeys were trained to move a custom made manipulandum in a horizontal plane (26 X 26 cm) with their right hand. This manipulandum moved a cursor on a computer screen placed 50 cm in front of the animal’s face. The monkeys initiated a trial by keeping the cursor inside a central green circle for a random period (1-1.5s, Figure 1). Two different targets (cues) were simultaneously displayed on the screen in two of the four possible directions relative to the center (0, 90, 180, and 270°) and the monkeys were free to select any direction of movement. The cues appeared randomly in any of four directions. The cues were chosen randomly for each session from a databank of 100 pictures and remained unchanged throughout the entire session. To induce a situation in which there was always a “better” choice, a single trial could not include two identical cues or two cues in the same location. Each cue was associated with a reward probability (provided that it was a motor successful trial) that remained constant throughout the session. We used 3 different pairs of reward probabilities for the two targets: (0.9 vs.0.6), (0.75 vs. 0.25) and (0.67 vs. 0.33). The target type and the reward schedule varied from session to session. After a random period (1–1.5 s), the disappearance of the black central circle indicated a “Go” signal, and the monkey initiated a movement toward one of the two targets. The cursor had to be maintained on the target for a random period in the range of 0.5–1 s, before being moved back to the central circle. To complete the trial, the monkey had to maintain the cursor inside the central circle for a minimum random duration (0.8–1.2 s). Disappearance of the central circle indicated to the animal that it had succeeded. If the animal completed the trial in due time and accurately enough, the reward was delivered (0.3 ml of fruit juice) according to the probability associated with the selected target. The trials were separated by 2–2.5 s inter-trial intervals (ITIs), during which the screen was black. In the case of an error, the trial was aborted, followed by an ITI. Once their motor success rate (i.e. the ratio of trial in which the animals completed the task without error) stabilized at 0.95 for a series of 200 trials, a recording chamber was implanted on the skull of each animal. The surgical procedure for attaching the recording chamber has been extensively described in a previous publication [22].

### Recording and data acquisition

A total of 61 behavioral sessions were recorded and analyzed in the behavioral part of our study. Within these sessions, 23 sessions (12 and 11 in monkey 1 and 2, respectively) were undertaken with multi-unit electrophysiological recordings. We recorded single unit activity of 192 left dorsal putamen neurons (78 and 114 in monkey 1 and 2, respectively). The procedures for multi-unit electrophysiological recordings, neural population sorting, and data acquisition have been described in a previous publication [20]. During the lowering of the electrode, the first neurons observed had a low tonic firing rate typical of cells in the putamen (0.5–5 spikes/s). We recorded extracellular spike activity of presumed projection neurons (medium spiny neurons (MSNs)), which showed very little spontaneous activity [23], although no such activity was detected with putative interneurons (tonically active (TANs) and fast spiking interneurons (FSIs)), which showed irregular tonic discharge [24]. We used 4 glass-coated Tungsten microelectrodes (0.5MOhm at 1kHz) lowered with a computer driven positioning system (Electrode Positioning System; Alpha Omega Engineering, Nazareth, Israel) until the typical signal of striatal neurons was detected. Signal was amplified with a gain of 10^3^ and filtered with a bandpass of 300 Hz to 6 kHz (Multi-Channel Processor; Alpha Omega Engineering). The electrical activity was sorted and classified on-line when the spike-to-noise ratio was approximately>3. Single unit sorting and classification were performed using a template-matching algorithm (Multi-Spike Detector; Alpha Omega Engineering). Data were stored by means of an analog-to-digital converter at 12 kHz (AlphaMap; Alpha Omega Engineering). After spike sorting, we averaged spike waveforms for each neuron by using the spikes collected during three minutes of each recordings and labelled neurons as putative MSNs, putative TANs or putative FSIs according to three features of the shape of the average waveforms: the length of the initial deflection, the length of the valley, and the sum of these two parameters [25]. Neurons were sorted as FSIs if their spikes displayed short total waveforms with negative initial deflections and neurons were sorted as TANs when their average spike presented long waveforms with positive initial deflection. The neurons that presented intermediate waveforms with negative initial deflections were sorted as MSNs. We also examined the firing rate and the coefficient of variation (CV=std/mean) of the interspike intervals collected for each neuron as two additional criteria for the classification. MSNs displayed low firing rates and variable coefficient of variations of the ISI ranging from 1 to 14 while TANs and FSIs typically displayed high firing rates and consistent coefficient of variations of the ISI. This study focuses only on MSNs for which the size of the data set was sufficiently large.

### Behavioral data analyses

 The analyses were performed with custom-made Matlab (MathWorks, Natick, MA) programs and NeuroExplorer (Nex Technologies, Littleton, MA). The results are expressed in the form of mean ± SEM. For behavioral data analyses, only successful motor trials were kept. All other trials were considered as error trials and were discarded from the databank used for successive analyses. Learning curves were constructed by averaging choices over sessions: for each session, we constructed a binary vector representing the successive choices of targets such that 1 corresponds to trials in which the target associated with the higher-probability of reward was chosen and 0 otherwise. Learning curves were smoothed with a Savitzky-Golay filter [26] with a window length of 11 trials. This filter was used because it removes high frequency noise from the data while preserving the main peaks.

Session type was determined by the value of the *preference* in the session, where the preference in a session is defined as the fraction of the last 50 trials in which the most rewarding cue was chosen. Sessions for which the preference was equal to or larger than 0.7 were defined as melioration sessions; sessions in which the preference was equal to or less than 0.3 were defined as minimizing sessions; All other sessions were defined as no-learning sessions. These thresholds were set because the probability of a no-learning session, assuming that choices are fair Bernoulli trials, is 99%.

In order to study direction selectivity, we constructed a Direction Selectivity Index (DSI):

$$DSI=\frac{\max(Q_{D})-\min(Q_{D})}{\max(Q_{D})}$$ (1)

where *Q*_*D*_ corresponds to the number of times that a particular direction *D* (0, 90, 180 or 270°) was chosen in the last 50 trials of the session. The higher the value of DSI, the stronger is the direction preference. The significance threshold, 0.68 was set such that the probability that a random selection of directions will result in direction preference is 5%. 

### Neural data analyses

The analyses were performed with custom-made Matlab (MathWorks, Natick, MA) programs and NeuroExplorer (Nex Technologies, Littleton, MA). Trials were sorted according to chosen cue and neural activity was analyzed during the period surrounding the onset of the cues. We defined the window of 1 second that precedes cues onset the “early decision period” and the window of 1 second that follows cues onset the “late decision period”. We reasoned that cues must be evaluated during the two periods adjacent to cues onset and defined the sum of these periods as the “decision period”. We further assumed that neurons implicated in the decision process must be active during the “late decision period” because it immediately precedes choices and thus restricted our analyses to the 113 neurons that displayed discarge rates higher than 0.5 spike/second after cues onset [19][1]. As a result we analysed 51 neurons from 11 melioration sessions, 24 neurons from 5 no-learning sessions showing no direction preference, 21 neurons from 4 no-learning sessions showing direction preference (called direction preference sessions). We discarded sessions that show minimization because samples were low (1 neurons from 1 minimization session showing no direction preference and 16 neurons from 2 minimization sessions showing direction preference). Average neural responses to task events were obtained using a Savitzky-Golay filter with a window length of 60 ms since this filter maintained average peak profile. The average neural activity registered during the “decision period” was compared between the chosen cue conditions for every neuron with a Wilcoxon signed rank test. Significance threshold was set at 5%.

 Neurons were then classified according to the dynamic properties of their response to the cues, conditioned on the choice of the animal. If no significant difference between the responses to the two cues was observed in the first 15 trials in which each cue was chosen but was observed in the last 15 trials the neuron was classified as Learning Cue Preferences (or L-neuron), reflecting the fact that preferences for the chosen cue emerged over time. If the firing rate in the first 15 trials was significantly different between cues but this significant difference disappeared in the last 15 trials, then the neuron was classified as Forgetting Cue Preferences (or F-neuron). If the firing rate in the first 15 trials was significantly different between cues, and this significant difference persisted throughout the entire duration of the recording (i.e., significant difference in the last 15 trials), the neuron was classified as responding to Initial Preference (hence referred to as IP-neuron). All *other* neurons, in which response was higher than 0.5 spikes/second during the “late decision period”, were considered as non-specific responding neurons (R-neurons).

 To investigate the information encoded by the Learner neurons we performed a linear regression between the difference of firing activity between cues recorded for each L-neuron and their corresponding session preference. The difference of firing activity was calculated from the 15 last trials in which each cue was chosen.

To investigate the role played by dorsal putamen population activity we performed a linear regression between the firing activities recorded for all active MSNs (n=88) averaged over the 3 first trials and their corresponding session preference. The 3 first trials are the earlier period that significantly predicted preferences (Pearson correlation, R=0.21, *p*<0.05). Regressions between firing activities in the first (or two firsts) trial(s) and session preferences were not significant (Pearson correlation, R=0.18, p=0.08 and R=0.17, p=0.10 respectively). Firing activity was then averaged over successive bins of 10 dorsal putamen neurons arranged in ascending order of firing activity (the last bin was only 8 neurons). The collected values were plotted with the average preferences created during the sessions in which the group of 10 neurons included in a bin was recorded. 

To ensure that comparisons of discharge activities between behavioral profiles were not confounded by direction preference, sessions in which monkeys showed both melioration, minimization or no-learning with direction preference were removed from the analyses.

### Theoretical reconstruction of recording sites aligned with putamen’s somatotopic maps

We reconstructed the recording sites on histological maps of the striatum ranging from -2 mm to +2 mm from the anterior commissure (AC). Recordings were pooled together for both monkeys on a template atlas of the macaque brain [27]. The somatotopic maps of the striatum described by Takada et al. [28], were drawn, overlaid then scaled to our maps of the striatum after alignment to the anterior commissure and scaled. We then drew the functional territories on the maps. 

## Results

 Two rhesus monkeys were trained to make repeated choices in a custom version of the two-armed bandit task [8-10,29], in which the locations of two cues were randomly assigned among four directions (Figure **1**, for detail, see method section). Each cue was associated with a reward probability that remained constant throughout the session but varied between sessions. We used 3 different schedules of reward probabilities for the two cues: (0.9 vs. 0.6, 0.75 vs. 0.25 and 0.67 vs. 0.33). 

### Monkeys’ learning curve

**Figure 2:**
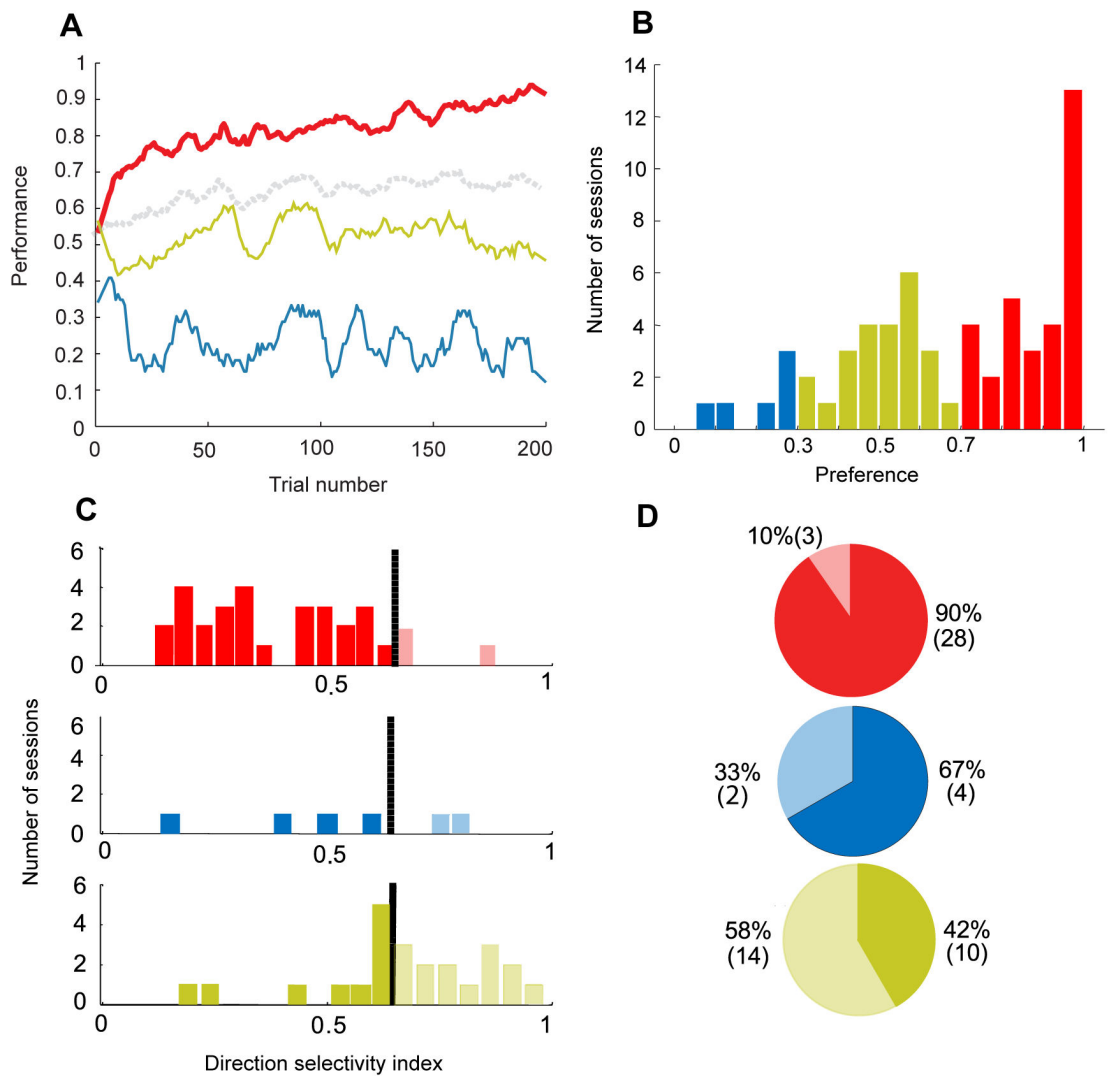
Learning behavior. (**A**) Average learning curves in all sessions (*n*=61, gray), melioration sessions (*n*=31, red), no-learning sessions (*n*=24, green) and minimizing sessions (*n*=6, blue). (**B**) Distribution of *preferences*, average choices in the last 50 trials of the session, for melioration, no-learning and minimizing sessions. (**C**) Distribution of direction selectivity indices (DSIs) for the different sessions. Black vertical solid line marks direction preference threshold. (**D**) Pie-chart representing the ratio of sessions showing direction preference (hatched) for each behavioral profile. Colors in B-D are as in **A**.

 Learning behavior is typically quantified by computing the learning curve, the fraction of trials in which the most rewarding cue was chosen as a function of time, averaged over several sessions. The learning curve, averaged over all sessions and both monkeys, depicted in Figure **2A** (dashed grey line), demonstrates that within several tens of trials, subjects formed a preference in favor of the most rewarding cue such that on average, the monkeys chose the most rewarding cue in 68% of the last 50 trials, which is significantly more than would be expected by chance (Figure **2A**, Mann-Whitney U test, *p*=10^-6^).

### Substantial variability between sessions

 The average learning curve presented in Figure **2A** (grey line) is similar to previously described learning curves [30-33]. However, it conceals the monkeys’ behavior in individual sessions. A more careful analysis revealed substantial variability between sessions. To quantify this variability, we considered, for each session, the fraction of trials in which the most rewarding cue was chosen in the last 50 trials of the session. We denote this fraction as the monkey’s *preference* in that session. The distribution of monkeys’ preferences, depicted in Figure **2B**, is wide, ranging from 10% to 100%. In 51% (31/61) of the sessions, the monkeys’ preference was significantly larger than chance (red in Figure **2B**), demonstrating that the monkey shifted its preference in the direction of the most rewarding cue. We denote these sessions as melioration sessions. In 55% (17/31) of the melioration sessions, melioration cue was chosen almost exclusively (in more than 90% of the trials). In the remaining 45% (14/31) of the melioration sessions, melioration cue was chosen between 70 and 90% of the trials. In 39% (24/61) of the sessions, the monkeys showed no cue-related preferences (green). We denote these sessions as no-learning sessions. Surprisingly, in 10% (6/61) of the sessions the monkey showed a marked preference towards the less rewarding alternative (blue). We denote these sessions as minimizing sessions. The three patterns are also evident when plotting the learning curves separately for the three groups of sessions (Figure **2A**, red, green and blue lines). Moreover, all three types of behavior were observed in both animals (Figure **S1A**) and for all reward schedules (Figure **S1B**), such that average preference was statistically similar between reward schedules (F(2, 58) = 1.02, p = 0.36, one-way ANOVA) and the proportions of each profile was also similar across reward schedules (p = 0.32, 2-tail Fisher’s exact test) demonstrating that this variability in learning is not the outcome of differences between the two monkeys or the differences in the reward schedule. 

### Directional preference

 In the reward schedules used, the locations of the cues on the screen were random and therefore the probability of reward was independent of location. Yet surprisingly, we found that in many sessions, monkeys developed a direction preference. To quantify this preference, we constructed a direction selectivity index (DSI) for each behavioral session (see Equation 1 in the Methods and Materials). We found that in 58% (14/24) of the no-learning sessions, in which there was no cue-related learning, monkeys developed a preference towards one of the directions (Figure **2C**, light colors). A smaller percentage of direction selectivity, 13% (5/37) was observed in the sessions in which monkeys developed cue-related preferences (melioration and minimization; Figure **2D**). The difference in the fraction of direction selective sessions between the learning and no-learning sessions can be attributed to the independence of the direction of a cue and its identity. If the monkey chooses one of the cues exclusively then as a result of independence of reward and location in the reward schedule, no direction preference is possible. Thus, the stronger the cue preference, the weaker is the maximally possible DSI.

### Variability in dorsal putamen subpopulations correlates with fluctuations in learning

The neural basis of learning behavior has been a subject of intense research in the last decade. Of particular interest to us is the involvement of the dorsal putamen, a major input nucleus of the basal ganglia. It has been previously shown that after learning, the firing rate of approximately 10% of striatum neurons is sensitive to the action values of the chosen cues [19,20,34-36], suggesting that the dorsal putamen plays an important role in the learning and the execution of the task. 

**Figure 3:**
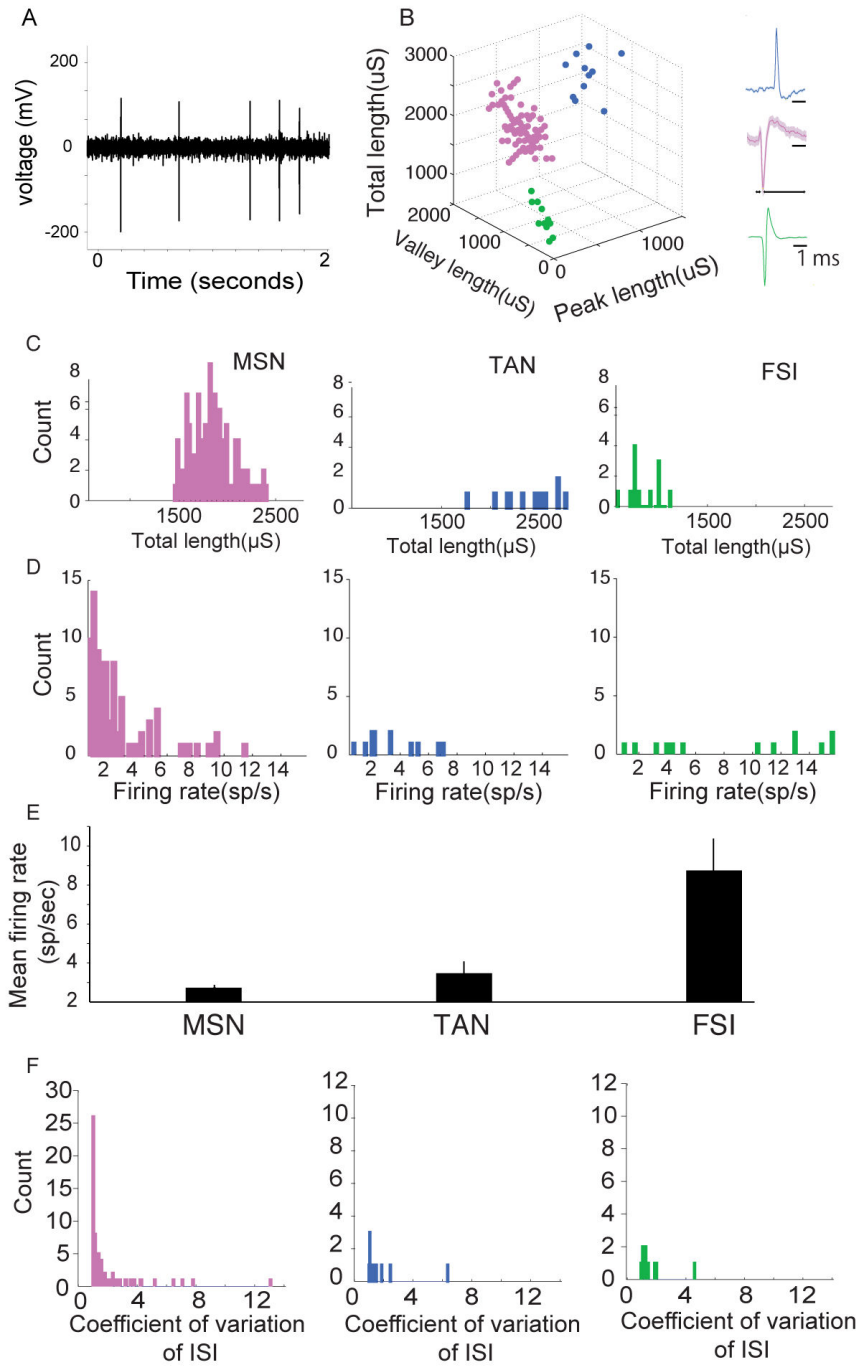
Characterization of cell subtypes. (**A**) Filtered signal obtained from extracellular recording of a dorsal putamen neuron amplified with a gain of 10^3^ (bandpass filter 300 Hz-6 KHz). (**B**) The parameters of mean waveforms for each neuron were plotted against each other, revealing three clusters. After spike sorting, the width of each phase of each spike was calculated. Spikes were then clustered using three parameters related to the length of the waveform: the length of the total negative deflections (Peak length), the length of the valley, and the sum of these two parameters (Total length). (**C**) Distribution of the waveform’s total length for the each type of neurons; color codes are as above. (**D**) Distribution of the firing rate of the neurons; color codes are as above. E, Population firing rate for the three cell subtypes. The firing rate of the interneurons was significantly higher than that of the MSNs. F, Distribution of the coefficient of variation (CV) of the interspike intervals of the three types of neuron. The interspike intervals of the presumed MSNs tend to be more variable than that of the presumed interneuron types.

 We recorded 192 neurons in the dorsal putamen (Figure **3**) of the two monkeys performing the task and focused our analyses on the 113 neurons (58.9%) that were significantly more active (firing rate >0.5 spikes/secondes) during the decision period. We sorted active neurons according to three cell types: the projection neurons (medium spiny neurons (MSNs), n=88), and the interneurons (tonically active neurons (TANs), n=12, and fast spiking interneurons (FSIs), n=13) (Figure **3**). The population of Putative MSNs produced spike waveforms typically longer than FSIs but shorter than TANs (Figure **3C**), displayed low firing rates (Figure **3D, E**) and tended to fire more phasically (wide range of ISI, Figure **3F**). Because of the small size of the dataset collected for putative TANs and FSIs, we restricted our analyses to putative MSNs. 

**Figure 4:**
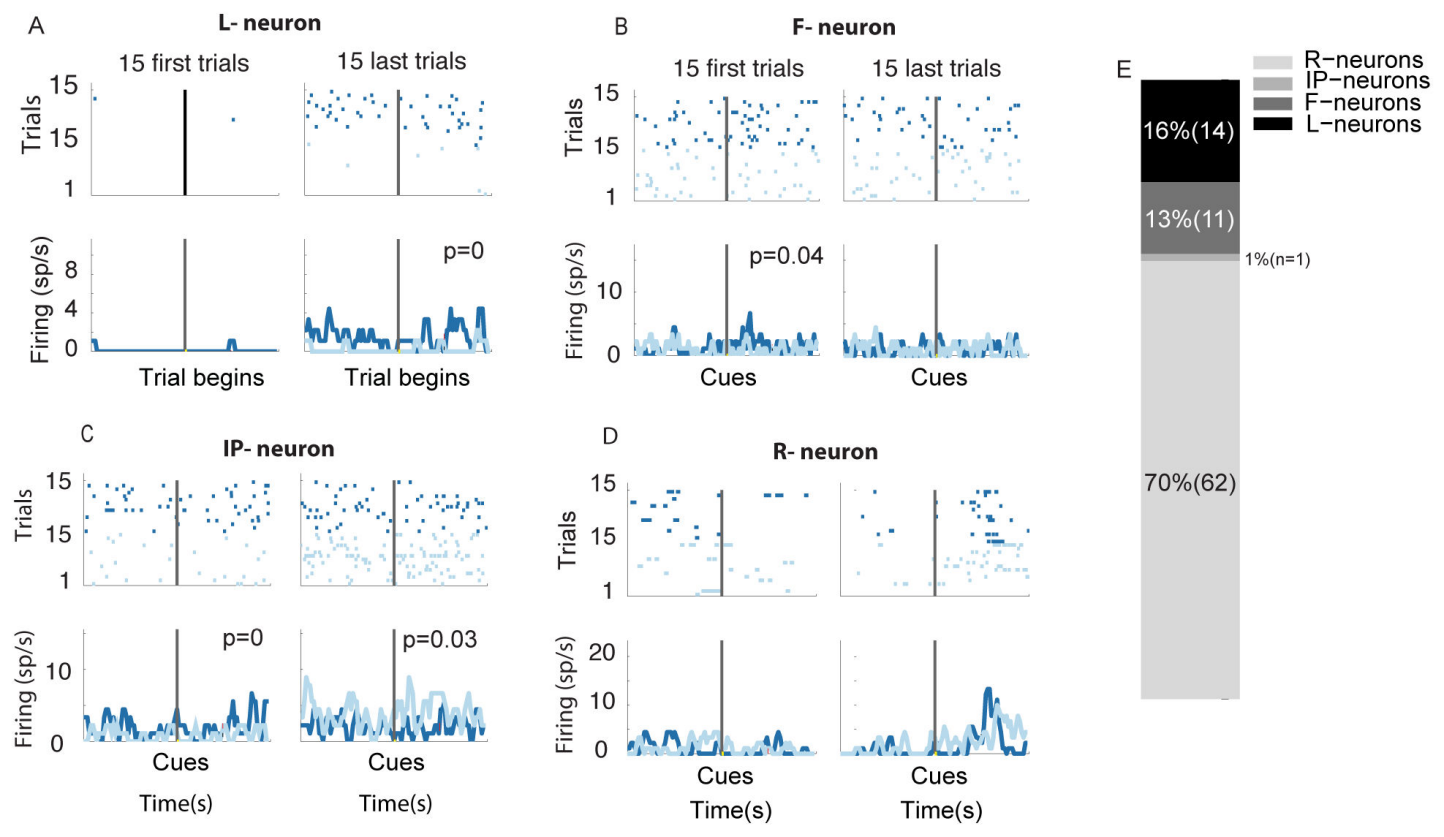
Neural activity at Single cell level. Perievent rasters and perievent histograms for an example (**A**) L-Neuron, B) F-Neuron, C) IP-Neuron and D) R-Neuron. The perievent rasters and perievent histograms are aligned either at the start of the trials or at cue onset, separately for trials in which most rewarding (dark blue) and less rewarding (light blue) cue was chosen. (**B**) Stacked bar charts of the distribution of the recorded neurons.

 Putative MSNs were then sorted according to their differential responses to the choices performed by the animal during an early and late decision period (windows of 1s before and 1s after cues onset respectively) and how this differential response evolves during the session (Figure **4A-D**). Population data are summarized in Figure **4E**. Among putative MSNs, we classified 1 neuron (1% of the active MSNs) as responding to initial preference (IP neurons, Figure **4C&E**). Another subset of 14 neurons (16% of the active MSNs) progressively fired differently in trials in which the different cues were chosen, suggesting that these neurons responded to preference for the chosen cue and the change in their behavior during the session reflects the learning of the values. We thus refer to these neurons as learning Cue Preference (L-neurons, Figure **4A&E**). Another 11 neurons (13% of the active MSNs) showed the opposite pattern, discriminating between cues early in the session but progressively loosing their selectivity, suggesting that they forget Cue Preference (F-neurons, Figure **4B&E**). The remaining 62 neurons (70% of the active MSNs) could not be discriminated according to the above-defined criterions (R-neurons, Figure **4D&E**). 

**Figure 5:**
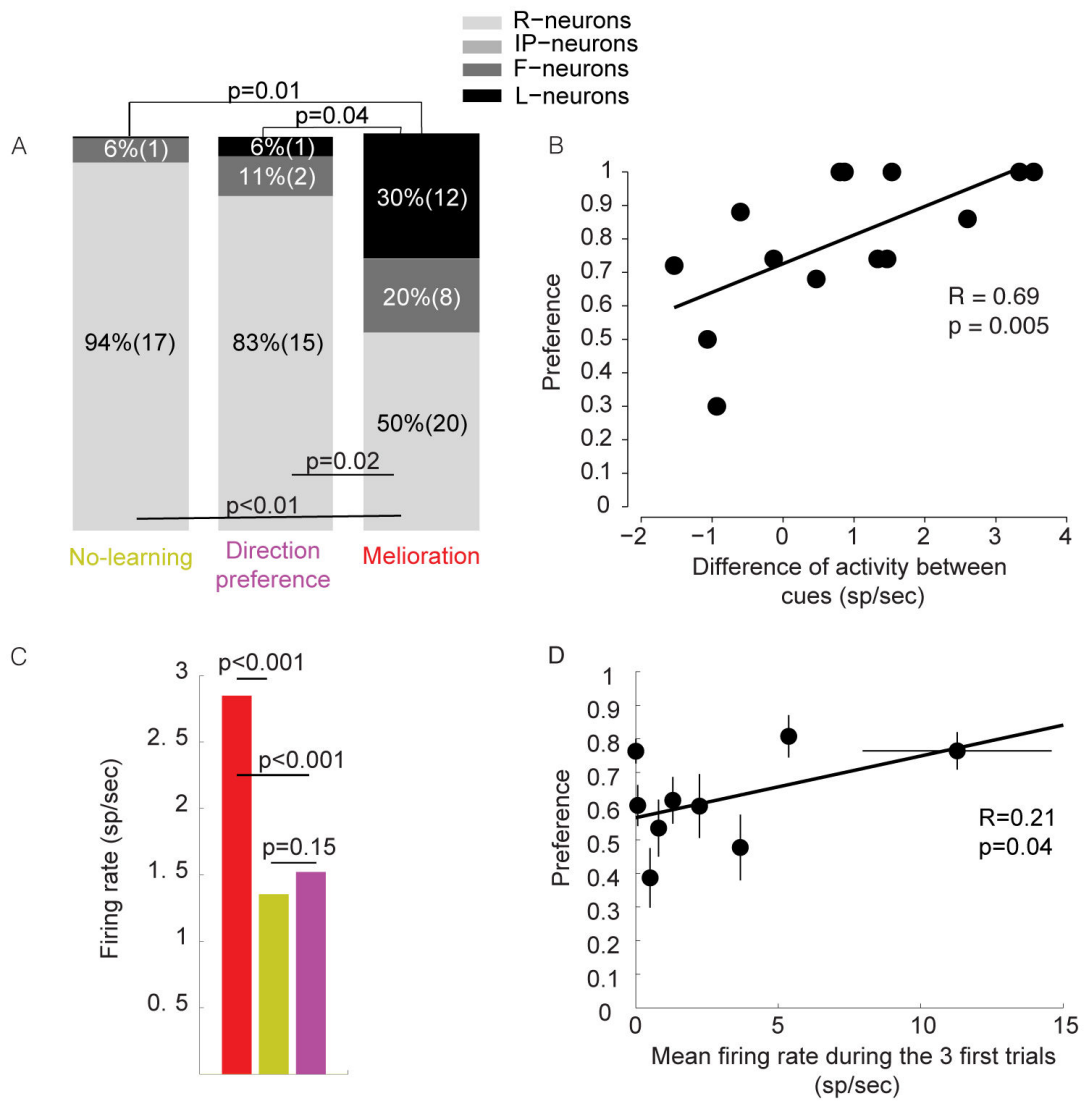
Neural activity at single cell and populations level. (**A**) Stacked bar chart of the distribution of the recorded neurons sorted according to behavioral profiles except minimization for which the dataset was too small (n=11 neurons). (**B**) The preferences calculated for the 9 sessions during which L-Neurons were recorded are plotted against the differential activity of the L-Neurons (n=14 neurons) for the two cues. Differential activity was calculated from the last 15 choices of each cue (15 trials for the best cue and 15 trials for the worst cue). (**C**) Population firing activity for each of the behavioral profiles except for minimization (mean±s.e.m; Wilcoxon rank sum test): melioration sessions (red, 11 sessions, 40 neurons); no-learning sessions (green; 5 sessions, 18 neurons); direction preference sessions (purple; 4 sessions, 18 neurons). For melioration, minimization and no-learning conditions, sessions with direction preference were discarded and direction preference condition corresponds to no-learning sessions in which monkeys showed preference for a direction (**D**) Average firing activity during decision period averaged over the 3 first trials as a function of preference (performance on the last 50 trials). Line is the least square error linear fit. Horizontal error bars indicate the SEM calculated over the 10 neurons contained in a bin (8 neurons in the last bin); Vertical error bars indicate the SEM calculated over the sessions that correspond to the neurons included in a bin.

If the dorsal putamen plays an important role in learning preferences for the cues and in utilizing these preferences in the process of decision-making then variability in the distribution of the different classes of neurons may result in variability in the ability to learn. For example learning may require that a high number of active MSNs learn Cue Preference. The prediction of this hypothesis is that a higher fraction of L-neuron/active neuron will be observed in melioration sessions than in other behavioral profiles. To test this hypothesis, we compared the fraction of IP, L, F and R-neurons between melioration (red in Figure **2B**) and no-learning sessions (green in Figure **2B**) and found no difference between melioration and no-learning in the fraction of IP neurons (0/40 vs. 0/18, *p*=1, two-tailed Fisher exact test, Figure **5A**), or F neurons (7/40 vs. 1/18, *p*=0.41, two-tailed Fisher exact test, Figure **5A**). By contrast, the fraction of L and R-neurons differed significantly between the two groups. While L-neurons composed 30% of the active MSNs during melioration sessions (12/40), no L-neuron were found (0/18) during no-learning sessions (*p*=0.01, two-tailed Fisher exact test, Figure **5A**). The proportion of R-neurons changed in the opposite direction. The fraction of R-neurons was lower (50%, 20/40) during melioration than during no-learning sessions (94%, 17/18; *p*<0.01, two-tailed Fisher exact test). Similar results were obtained when comparing melioration and direction preference sessions. Only 6% of active MSNs (against 30% during melioration, p=0.04, two-tailed Fisher exact test) were L-neurons while 83% (against 50% during melioration, p=0.02, two-tailed Fisher exact test) were R-neurons when monkeys showed direction preference. Finally the fraction of R and L-neurons should not change for comparisons that do not involve learning. Accordingly, comparing No-learning and direction preference, we found no significant difference between IP-neurons (0/18 vs. 0/18, p=1, two-tailed Fisher exact test), L-neurons (0/18 vs. 1/18, p=0.99, two-tailed Fisher exact test), F-neurons (1/18 vs. 2/18, p=0.99, two-tailed Fisher exact test) or R-neurons (17/18 vs. 15/18, p=0.6, two-tailed Fisher exact test, Figure **5A**). Thus learning correlates with balance of L/R-neurons and changes in this balance is correlated with maladaptive behaviors. 

### The intensity of encoding of cue-preference by L-neurons predicts fluctuations in learning

Learning could also change with the intensity with which neurons encode Cues Preference such that behavioral preference increase as L-neurons fire more for one cue than for the other. We tested this hypothesis by regressing the differential firing activities of L-neurons between cues with preference. The 15 first trials in which the most or least rewarding cue was chosen were separated and firing activities were substracted. Then differences in activities between cues were regressed with corresponding preferences. We found that the differential activities of L-neurons between cues were strongly predictive of monkeys’ ability to learn (Spearman correlation, p=0.005, R=0.69, Figure **5B**) suggesting that learning also correlates with the intensity with which L-neurons encode cue-preference.

 However, despite the fact that a subset of the neurons encoded cue-preference (L-neurons), and that the intensity with which L-neurons encode cue-preference strongly predicted learning we found no statistical difference between the population activity in trials in which the most rewarding cue was chosen and trials in which the less rewarding alternative was chosen (*p*=0.1, Wilcoxon signed rank test). This is probably due to the fact that the contribution of L-neuron to the average activity was averaged out because of the variability in the responses of the various sub-populations. 

### Dorsal putamen neural population activity predicts fluctuations in learning

Surprisingly, the population average activity was correlated with the *ability* to learn: dorsal putamen neurons were twice more active during sessions in which monkeys developed a significant preference towards melioration alternative, compared with the other two patterns of behavior (*p*<0.001, Wilcoxon rank sum test; Figure **5C**). 

Moreover, the difference between melioration and non-learning trials is already significant at the very beginning of the block: population averaged firing activity during the *first* 3 trials was significantly correlated with the primates performances during the *last* 50 trials of the sessions (R=0.21, *p*<0.05, Figure **5D**), demonstrating that the activity in the dorsal putamen in the very early trials predicts the ability of the animal to learn to prefer the most rewarding cue. This result suggests that dorsal putamen may contribute to learning at three different levels. While a specific dorsal putamen’s neuronal subpopulation enables the representation of cue preferences and the intensity with which it is encoded, dorsal putamen’s overall population is directly related to the subject’s ability to create a preference for the most rewarding cue and therefore increase reward rate.

### Neural responses were not somatotopically organized

**Figure 6:**
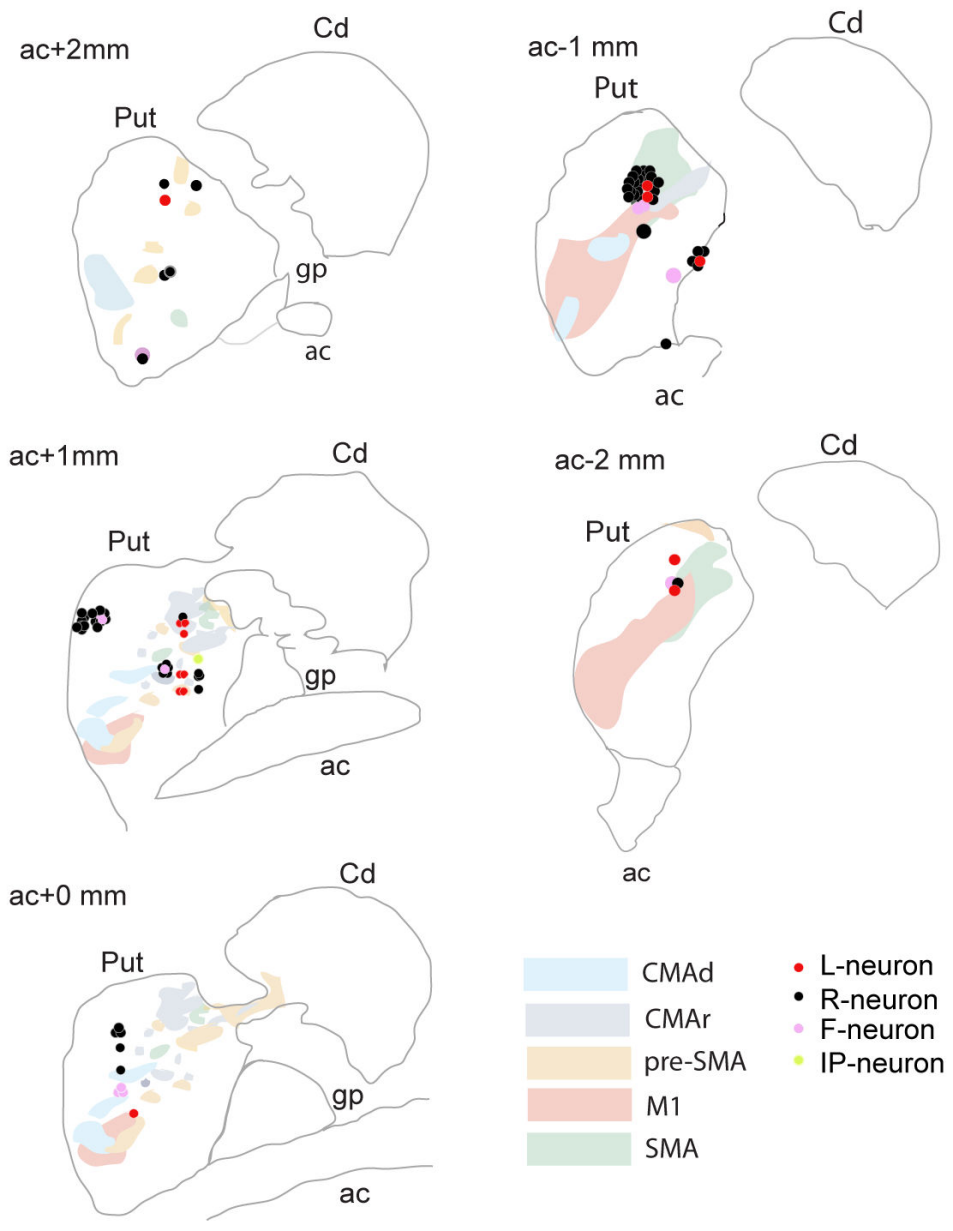
Reconstruction of the recording sites. The dots show the estimate locations of the electrodes tip during recording sessions (scale in mm). The recording sites of monkey 1 and 2 have been pooled. The colored areas correspond to the sensorimotor territories of the putamen identified in Takada et al[28]. *CMAd: dorsal* *cingulated* *motor* *area, CMAr: rostral* *cingulated* *motor* *area, pre-SMA: pre-supplementary* *motor* *area, M1: primary* *motor* *cortex, SMA: supplementary* *motor* *area*.

We then asked if L-neurons were located in specific regions of the putamen. Figure 6 show that all recordings were situated in the regions of the rostral and caudal sensorimotor areas of the putamen (anterior and posterior to the anterior commissure (AC) respectively [37]) in the zone of convergence of orofacial, forelimb and hindlimb regions of MI and SMA in the central zone of the dorsomedial-to-ventrolateral portion of the putamen and in the central zone of the dorsomedial-to-ventrolateral portion of the putamen which receives projections essentially from CMAr in rostral part, but also from pre-SMA and SMA at the location of the anterior commissure [28,37,38]. Those projections are segregated [28,37,38]. We didn’t record from limbic territories (ventral putamen), associative territories (essentially caudate and very rostral putamen at about +3mm from AC) or from areas receiving projections from dorsal anterior cingulate cortex (dACC, limbic areas) nor dorsolateral prefrontal cortex (dLPFC, associative areas [39]) that is more rostral[40]. Finally, the neural subtypes identified (L, IP, F and R-neurons) do not seem to respect any particular organization within the set of sensorimotor areas of the putamen.

## Discussion

In this paper we demonstrated substantial variability in operant learning. While in some sessions the monkeys learned to prefer the most rewarding cue, in other sessions they failed to learn, often forming irrelevant or detrimental preferences. At the neuronal level, this variability in behavior is correlated with variability in the distribution and intensity of cue-selective responses of individual neurons. In particular, the ability to learn is correlated with the existence of a large enough subpopulation of dorsal putamen neurons that acquire a preference for one of the two cues. At the population level, the firing rate of dorsal putamen neurons at the very *beginning* of the session is correlated with choice preference at the *end* of the session. These results reveal independent neural correlates for the acquisition of preferences. 

Previous studies have already shown that in two-armed bandit reward schedule tasks, subjects develop a preference towards the most rewarding cue yet fail to choose that alternative exclusively even after a long period of time [8-12]. It has been suggested that this failure to maximize reflects continuous exploration [18,41,42] which is consistent with the behavior we observed (gray curve in Figure **2A**), However the individual session analysis reveals that the averaged learning curve (dashed grey line) is not representative and does not reflect the behavior of the monkeys in the *individual* sessions. In a substantial fraction of the trials, the monkeys formed a strong preference and chose one of the alternatives almost exclusively, whereas in others no preference was formed, irrelevant directional preference or even preference towards the less rewarding alternative was formed (Figure **2A, B**).

The differences between the individual learning curves and the averaged learning curve bears similarities with a study by Gallistel and his colleagues showing that the gradual increase in performance observed in each individual subject may be an artifact of group averaging [43]. Thus, our results reinforce the concept that methods based on a session-by-session analysis provide new insights into the understanding of behavior. The novelty of the behavioral results presented in this study relies on the fact that we show that variability is within subjects and not only between subjects.

In the second part of the study we investigated the neural basis of these behaviors. We focused our attention on the dorsal putamen, which has previously been suggested to play an important role in decision making and learning. We found that 16% of active dorsal putamen neurons, supposedly MSNs, learn to encode preference for cues (L-neurons; Figure **4E**). This finding can be related with previous studies that demonstrated that the firing rate of a small fraction (10 to 20%) of dorsal putamen neurons is selective to the chosen cue [19,20,34-36]. Interestingly, we found several links between the fraction of L-neurons and the ability to learn to meliorate. We hypothesize that these links may reflect processes involving competition between prior preferences for cues and acquired preferences for cues driven by rewards [44]. In this framework, if the fraction of L-neurons in the population is not sufficiently large, prior preferences dominate choice, precluding learning. Most interesting, another dimension emerges when considering the firing rate of the neurons at the population level. dorsal putamen’s population activity predicts the ability to develop a preference towards the most rewarding cue (Figure **5C**). In other words, while at the individual level some neurons encoded cue preference, at population level dorsal putamen activity is correlated with the *ability* to learn in a session, predicting whether the monkey will have a "good" or "bad" learning day. This suggests that a general, population-wide increase in the firing rate of dorsal putamen neurons enhances the final performance of the animal. Our finding could be related to recent fMRI studies in human showing that the activity in the dorsal striatum at the beginning of a learning task is predictive of the final performances of the subjects [45][2]. 

Whether fluctuations in putamen’s activity and learning ability reflect changes in attentional or motivational processes is hard to distinguish. It is well-known that MSN neurons are generally silent at rest [46][3]. In this state they are not sensitive to cortical inputs unless these inputs are strong. However, in the presence of a strong-enough input, such as an attentional gain, these neurons become sufficiently active to be responsive to weaker modulations in cortical inputs [47][4]. Therefore, if the population of dorsal putamen neurons is in the low activity state, in case of low attention, the neurons are less able to process cortical information, which hinders learning. 

It is also known that dopamine release increases the firing rate of striatal neurons and as a result, increases their sensitivity to cortical inputs [48-50]. Previous studies have shown that dopamine is released during learning [51-53][5-7]. In addition to the other roles of dopamine as representing reward-related information, dopamine also gates the ability of the dorsal putamen neurons to respond to cortically-mediated information [54][8], which is necessary for the learning of the values of the actions. Thus, fluctuations in the overall levels of dopamine, which could be related to change in the motivational state [55] could result in fluctuations in the ability to learn to prefer the most rewarding cue. High concentrations of striatal dopamine increase the sensitivity of putamen neurons for cortical inputs such that the number of dorsal putamen neurons that are sensitive to reward-predictive cues and their sensitivity will increase. This dopamine-induced motivational gain favors stronger and better representations of reward-predictive cues over prior preferences relayed by competing cortical inputs, biasing the competition between reward-driven preferences and priors in efferent regions of the striatum in favor of the former. One could test this hypothesis by locally injecting dopamine antagonists in monkeys’ dorsal putamen at the very early trials of learning. The resulting impairment of dopamine transmission should mimic low motivational states and disrupt monkeys’ ability to learn, giving rise to maladaptive preferences.

All our recording were localized in the sensorimotor and motor planning areas of the putamen [37]. Some of those areas have been shown to be zones of convergence of orofacial, forelimb and hindlimb regions of MI and SMA in the central zone of the dorsomedial-to-ventrolateral portion of the putamen [28,37,38]. The other areas located in the central zone of the dorsomedial-to-ventrolateral portion of the putamen have been shown to receive projections essentially from CMAr in rostral part, but also from pre-SMA and SMA at the location of the anterior commissure. Those projections have also been shown to be segregated [28,37,38]. We didn’t record from ventral putamen (limbic territories), associative territories (essentially caudate and very rostral putamen) or from areas receiving projections from dorsal anterior cingulate cortex (dACC, limbic) nor dorsolateral prefrontal cortex (dLPFC, associative; [39]) more rostral [40]. These results suggest that the learning mechanisms identified occur in the sensorimotor and motor planning areas of the striatum that are known to be involved in decision making and habit-based behavior. This further suggests that the learning mechanisms identified may be the bases of decision-making processes driven by acquired habit-based preferences.

 In conclusion, our results showed that failure to meliorate and the formation of irrelevant or even detrimental preferences is reflected at the cellular level. These preferences may result from insufficient engagement of brain regions involved in learning processes. We hypothesize that the attention/motivational deficit leads to decreased dorsal putamen activity at the start of the session, which disrupts reward-based learning in cortico-basal ganglia circuits. Testing this hypothesis awaits further studies, as does an explanation for the formation of direction preference.

## Supporting Information

Figure S1 **Variability holds for both monkeys and all reward schedules.** (**A**) Distribution of preferences, average choices in the last 50 trials of the session, for no-learning (red), no-learning (black), and minimizing (blue) sessions, for monkey 1 (top) and monkey 2 (lower). (**B**) Distribution of preferences for the 3 reward schedules: bottom (0.9 vs. 0.6), middle (0.67 vs. 0.33) and top (0.75 vs. 0.25), for no-learning (red), no-learning (black), and minimizing (blue) sessions.
(TIF)
