# Supplementary figures and images for: Complex Population Response of Dorsal Putamen Neurons Predicts the Ability to Learn
Source: PLoS One. 2013 Nov 14;8(11):e80683. doi: 10.1371/journal.pone.0080683 (PMC3828263; doi:10.1371/journal.pone.0080683)

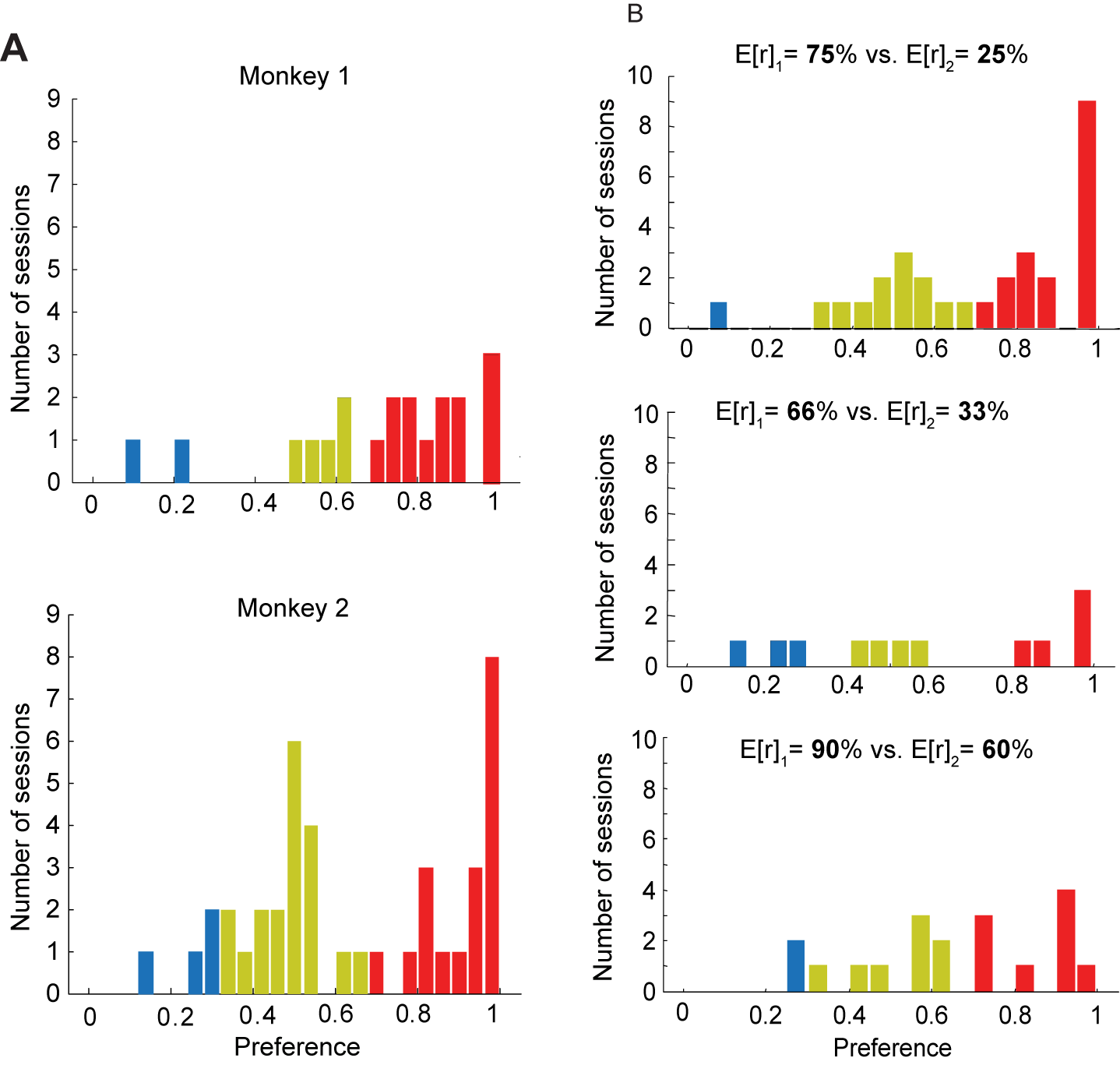

Supplement: Figure S1 — Variability holds for both monkeys and all reward schedules. (A) Distribution of preferences, average choices in the last 50 trials of the session, for no-learning (red), no-learning (black), and minimizing (blue) sessions, for monkey 1 (top) and monkey 2 (lower). (B) Distribution of preferences for the 3 reward schedules: bottom (0.9 vs. 0.6), middle (0.67 vs. 0.33) and top (0.75 vs. 0.25), for no-learning (red), no-learning (black), and minimizing (blue) sessions. (TIF) [file pone.0080683.s001.tif]
